# CD3xHER2 bsAb‐Mediated Activation of Resting T‐cells at HER2 Positive Tumor Clusters Is Sufficient to Trigger Bystander Eradication of Distant HER2 Negative Clusters Through IFNγ and TNFα

**DOI:** 10.1002/eji.202451589

**Published:** 2025-04-03

**Authors:** Chen‐Yi Liao, Patrick Engelberts, Michiel van Dijk, Annemieke Timmermans, John W. M. Martens, Elsa Neubert, Erik H.J. Danen

**Affiliations:** ^1^ Leiden Academic Centre for Drug Research Leiden University Leiden the Netherlands; ^2^ Genmab B.V. Utrecht the Netherlands; ^3^ Department of Medical Oncology Erasmus MC Cancer Institute Erasmus University Medical Center Rotterdam the Netherlands

**Keywords:** bispecific antibodies, bystander killing, cancer immunotherapy, T‐cells

## Abstract

Bispecific antibodies (bsAbs) bridging CD3 on T‐cells to tumor‐associated antigens (TAA) on tumor cells can direct T‐cell immunity to solid tumors. “Bystander killing”, where T‐cell targeting of TAA‐positive tumor cells also leads to the eradication of TAA‐negative cells, may overcome TAA heterogeneity. While bystander activity of activated, engineered T‐cells has been shown to be robust and wide‐reaching, spatiotemporal aspects of bsAb‐mediated bystander activity are unresolved. Here, we developed a model where breast cancer tumoroids varying in HER2 expression were printed in to extracellular matrix (ECM) scaffolds. We generated (1) mixed tumors containing different ratios of HER2^+^ and HER2^−^ tumor cells, and (2) HER2^+^ and HER2^−^ tumoroids spaced at different distances within the ECM scaffold. Subsequently, tumors were exposed to peripheral blood‐derived T‐cells in the presence of CD3xHER2 bsAbs. We find that CD3xHER2 bsAb‐mediated interaction of resting, nonactivated T‐cells with HER2^+^ tumor cells is sufficient (1) to eliminate 50% HER2^−^ cells in mixed tumor areas, and (2) to eradicate distant HER2^−^ tumor areas. Such bystander killing involves paracrine IFNγ and TNFα activity but does not require T‐cell accumulation in HER2^−^ areas. These findings indicate that bystander eradication of TAA‐negative cells can significantly contribute to bsAb therapy for solid tumors.

## Graphical abstract

Spatiotemporal aspects of bystander killing triggered by bispecific antibodies: (1) Resting T‐cells recognize HER2+ tumors via CD3xHER2 bsAbs. (2) Local T‐cell activation induces direct HER2^+^ tumor killing and IFNγ/TNFα release. (3) IFNγ/TNFα mediate paracrine killing of HER2^−^ cells. (4) HER2^−^ cells are eliminated in mixed (A) and distant (B) areas.

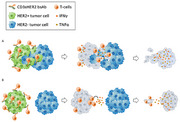

**Abbreviations**
bsAbs: bispecific antibodies
CM: conditioned medium
ECM: extracellular matrix
TAA: tumor‐associated antigen
TME: tumor microenvironment

## 1 Introduction

Cancer‐related mortality is caused in most cases by the propensity of the disease to spread to distant organs and its resistance to therapies. For patients, where local surgery does not eradicate the disease and radio‐ or chemotherapy faces severe side effects and resistance, prognosis is poor. Over the past few decades, cancer immunotherapy has provided new—and in some cases very promising—therapeutic options. Cancers develop mechanisms to escape immune surveillance through various means, such as downregulating antigen presentation, secreting immunosuppressive factors, and stimulating regulatory cells in the tumor microenvironment (TME) that dampen immune responses. Cancer immunotherapy involves strategies to overcome such escape mechanisms and trigger the immune system to recognize and kill cancer cells.

These strategies include the application of cytokines such as interferon‐gamma (IFNγ) to boost antitumor immunity [1] or immune checkpoint inhibitors that have shown efficacy in patients with hematological cancers or advanced cutaneous melanoma, significantly improving survival rates [2]. Also, adoptive cell therapies have been developed using patient‐derived tumor‐infiltrating T‐cells [3] or peripheral blood‐derived T‐cells genetically modified with engineered T‐cell receptors (TCR) selected for high affinity against a tumor‐associated antigen (TAA) [4]. These approaches depend on TCR recognition of TAA peptides presented by the MHC‐II complex on tumor cells.

Strategies that bypass MHC restriction include adoptive CAR T‐cell therapy, which has proven successful in treating certain hematologic malignancies [5], or bispecific antibodies (bsAbs) that bridge the CD3 antigen on T‐cells to a TAA on tumor cells. Such bsAbs trigger the formation of an immune synapse that is similar to that formed by natural TCR‐MHC/TAA interactions leading to T‐cell activation [6, 7]. Formation of the synapse triggers T‐cell‐mediated cytotoxicity mainly through the perforin‐granzyme B pathway or through the death receptor (Fas) pathway [8, 9]. In addition, the activated T‐cells produce IFNγ, which is secreted into the immune synapse for restricted activity [10] and also spreads and acts on nonantigenic bystanders [11].

IFNγ stimulates Fas expression on the tumor cells, rendering them more sensitive to contact‐dependent killing by T‐cells or other cells expressing Fas ligand (FasL). Additionally, it has been shown to have cytostatic or cytotoxic effects arresting or eliminating tumor cells independent of Fas‐FasL signaling [1]. Indeed, computational modeling has indicated that besides contact‐dependent T‐cell cytotoxicity, inhibition of tumor cell proliferation caused by IFNγ plays a major role in the ability of T‐cells to arrest cancer progression [12]. Moreover, it has been shown that the interaction of TCR‐engineered T‐cells with antigen‐positive tumor regions triggers IFNγ receptor signaling in antigen‐negative tumor cells at considerable distances in vivo [13, 14].

In mouse models, “bystander” eradication of antigen‐loss variants in solid tumors contributes to T cell‐based cancer immunotherapy [15, 16, 17]. Bystander killing of TAA‐negative tumor cells has been reported to involve tumor necrosis factor (TNF) and IFNγ [18, 19]. IFNγ appears critical for bystander killing in the context of CD3xTAA bsAbs [20, 21]. The mechanisms underlying CD3xTAA bsAb‐induced bystander killing are incompletely understood. While some studies report that bystander killing triggered by CD3xTAA bsAbs also requires Fas‐FasL signaling [19, 22] others have excluded the involvement of Fas [23, 24]. It is not known whether CD3xTAA bsAb‐mediated interaction of resting, nonactivated T‐cells with TAA‐positive tumor cells is sufficient to trigger effective bystander killing of TAA‐negative areas in tumors. The tolerance for the relative amount of TAA‐negative tumor cells in mixed areas and the reach of bystander killing from TAA‐positive to TAA‐negative areas in solid tumors are also unresolved for bsAbs.

Here, we developed a model where HER2‐expressing breast cancer tumoroids were printed in an extracellular matrix (ECM). In ∼40% of breast cancer patients, HER2 has been observed to be heterogeneously expressed [25, 26, 27]. Heterogeneity can present as distinct tumor islands differing in HER2 expression or as intermingled HER2^+^ and HER2^−^ tumor cells [25]. We mimicked these scenarios by generating (1) mixed tumors with different ratios of HER2^+^ and HER2^−^ tumor cells, and (2) HER2^+^ and HER2^−^ tumoroids spaced at different distances within the ECM scaffold. Subsequently, these models were exposed to resting, nonstimulated peripheral blood‐derived T‐cells in the presence of CD3xHER2 bsAbs, and spatiotemporal characteristics of bystander killing were determined.

## 2 Results

### 2.1 Development and Image Analysis of HER2 Mosaic ECM‐Embedded 3D Tumoroids

**FIGURE 1:**
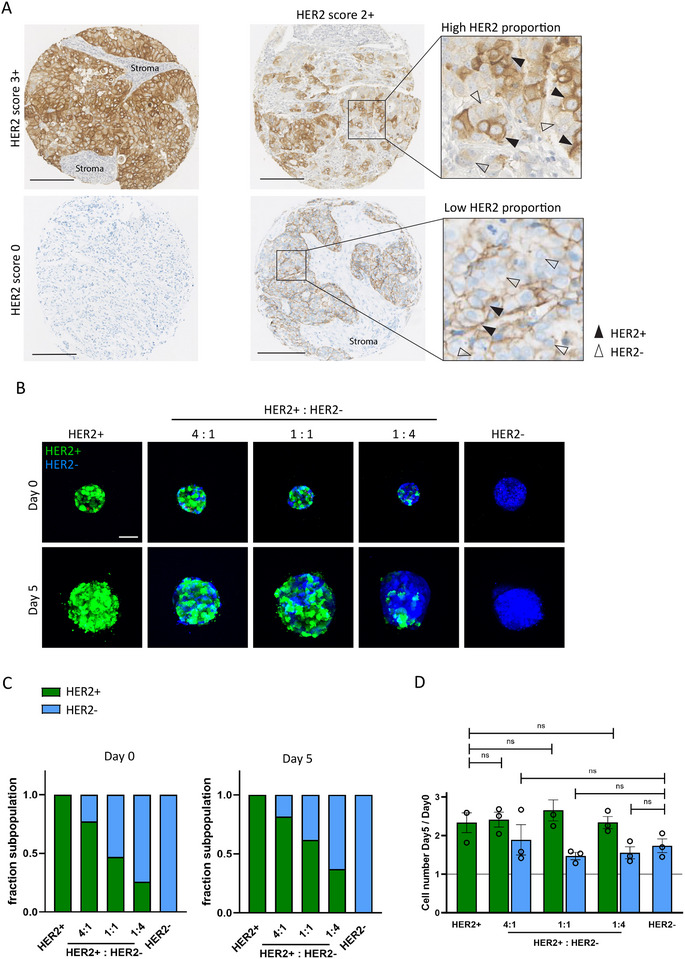
Development of HER2 mosaic ECM‐embedded 3D tumoroids. (A) IHC showing different levels of HER2 expression (brown) in human breast cancer sections. Black triangle = HER2^+^ cancer cells. White triangle = HER2^−^ cancer cells. The HER2 score was determined by pathologists at Erasmus Medical Center according to HercepTest guidelines. Scale bar = 200 µm. (B) 3D confocal imaging of ECM embedded HER2 mosaic tumoroids at day 0 and day 5 after BT474 cell cluster injection. HER2^+^ cells (BT474 WT, Lifeact‐EGFP‐transduced, green) and HER2^−^ cells (BT474 HER2 KO, Hoechst33342‐labeled, blue) were mixed at the indicated ratios and 100% HER2^+^ and 100% HER2^−^ tumoroids were used as controls. The images show maximum projections. Scale bar = 100 µm. (C, D) Quantification of confocal imaging data shown in (B). (C) Fractions of HER2^+^ and HER2^−^ cells in (mosaic) tumoroids on day 0 and day 5. (D) Fold change in numbers of HER2^+^ and HER2^−^ cells in (mosaic) tumoroids at day 5 calculated relative to day 0. Values above 1 indicate cell proliferation. The graph represents three independent experiments, each performed with one co‐culture per condition. Mean ± SEM is shown. Two‐way ANOVA followed by Tukey's multiple comparisons test was performed. ns = nonsignificant.

We examined HER2 staining in human breast cancer tissues and observed areas where HER2^+^ and HER2^−^ cells are intermingled (Figure 1A). To model such HER2 intratumoral heterogeneity, we generated mosaic tumoroids containing a mixture of HER2^+^ and HER2^−^ tumor cells. For this, we used a conditional HER2 knockout (KO) BT474 model using doxycycline‐inducible Cas9 and *ERBB2* sgRNAs (Figure S1A,B). We then asked to what extent HER2^−^ tumor cells within a 3D ECM‐embedded mosaic tumoroid are subject to bystander killing in the context of exposure to T‐cells and CD3xHER2 bsAbs. We verified that deletion of HER2 did not affect cell proliferation in standard 2D cultures (Figure S1C). To discriminate between HER2^+^ and HER2^−^ cells within mosaic tumoroids, HER2^+^ cells were transduced with pLenti‐Lifeact‐EGFP, while HER2^−^ cells were labeled with Hoechst. HER2 surface expression and cell proliferation in 2D culture were not significantly affected by Lifeact‐EGFP expression (Figure S1D–F).

To generate mosaic tumors with varying ratios of HER2^+^ and HER2^−^ cells, Lifeact‐EGFP transduced BT474 cells and Hoechst33342‐labeled BT474 HER2 KO cells were mixed at ratios of 4:1, 1:1, and 1:4. Droplets of the mixtures were injected into multi‐well plates prefilled with collagen I matrices to form tumoroids. Tumoroids consisting of 100% HER2^+^ or 100% HER2^−^ cells were used as controls (Figure 1B; Figure S2A). The ECM‐embedded tumoroid cultures were maintained for 5 days and analyzed by automated confocal fluorescence microscopy (Figure 1B). The fractions of HER2^+^ and HER2^−^ cells in the tumoroids were determined by quantifying the number of green and blue cells in each z‐plane and data were summarized for 3D analysis (note that images show projections while quantitative 3D image analysis was performed by combining data from each confocal section).

To establish a quantitative analysis of the relative response of HER2^+^ and HER2^−^ cells to cytotoxic events in this setup, tumoroids were treated with cisplatin, and propidium iodide (PI) was added to label dead cells. The amount of green and blue cells and PI‐positive cells were analyzed after 5 days. Background cell death in HER2^+^ and HER2^−^ tumoroids was negligible whereas near complete PI staining of tumoroids was observed in the presence of cisplatin, and, in agreement, a ∼90% reduction in green and blue fluorescence signals was observed (Figure S2B,C). The HER2^+^:HER2^−^ ratios as determined by microscopy at day 0 (2 h after tumoroid printing in the collagen matrix) matched the input ratios (Figure 1C). Only at day 5, a slight increase in the proportion of HER2^+^ cells was detected in the tumoroids. During the 5‐day tumoroid culture period, the number of HER2^+^ cells increased >twofold in the various tumoroids (Figure 1D; Figure S2C). For HER2^−^ cells this was ∼1.7‐fold, indicating some growth suppression in 3D tumoroids that was not observed in 2D culture. The slightly reduced growth of HER2^−^ tumoroids was not due to the cytotoxic effects of the Hoechst33342 dye used to label HER2^−^ cells, as HER2^−^ tumoroids exhibited slower growth compared with HER2^+^ cells and similar background PI staining, also when both cell types were labeled with Hoechst33342 (Figure S2D, E).

These results demonstrated a setup with a satisfactory assay window for quantitative analysis of the impact of T‐cell/ bsAb combinations on the relative growth and survival of TAA^+^ and TAA^−^ cells in ECM‐embedded tumoroids.

### 2.2 Determining the Proportion of TAA^−^ Tumor Cells in a Mosaic Tumor That Is Compatible with Complete Tumor Destruction Through Bystander Killing

**FIGURE 2:**
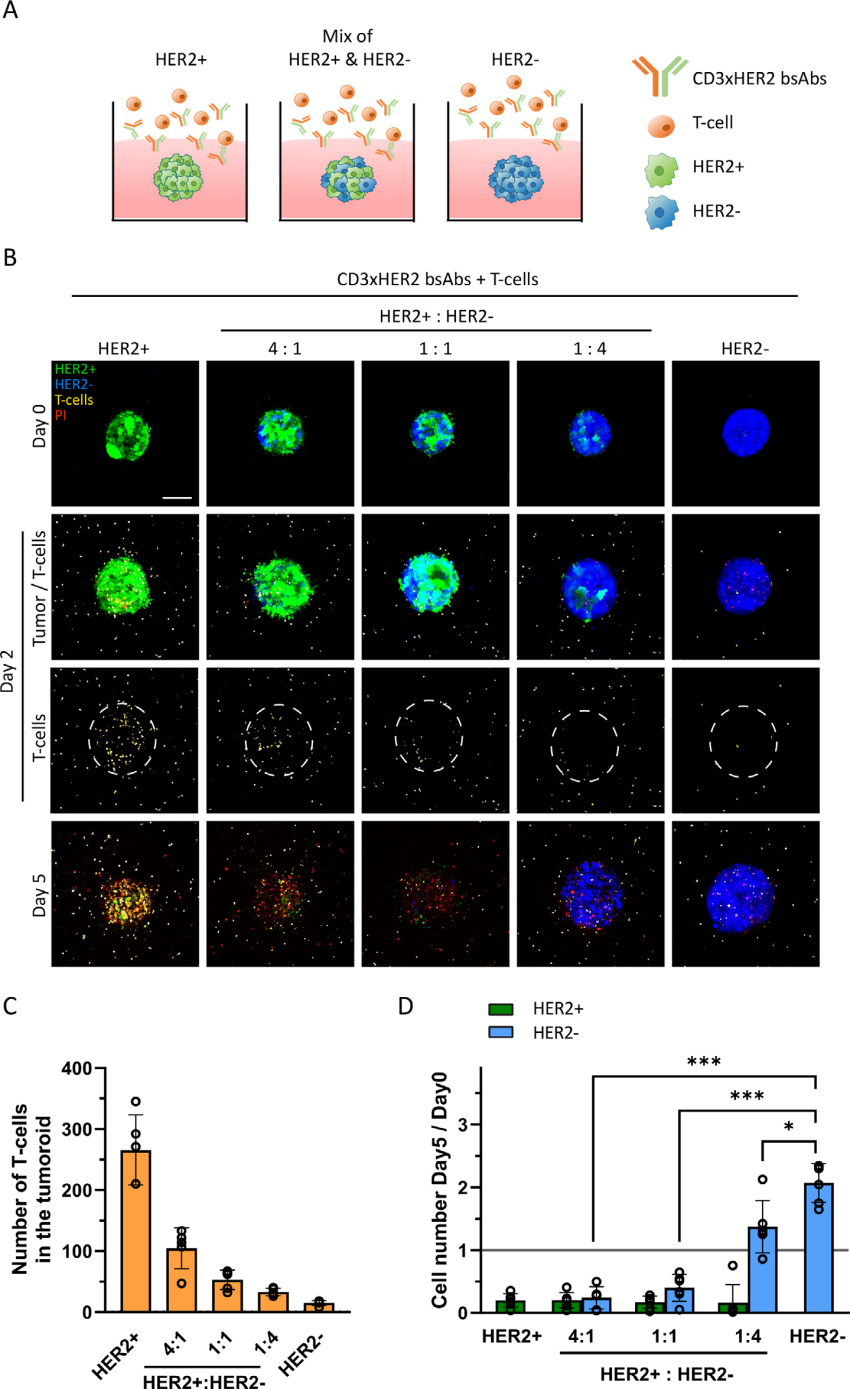
Proportion of HER2^−^ cells in mosaic tumoroids compatible with bystander killing. (A) Schematic representation of the experimental setup to investigate bystander killing. (B) Maximum projections of 3D confocal imaging of 100% HER2^+^, 100% HER2^−^, and HER2 mosaic tumoroids with the indicated HER2^+^:HER2^−^ ratios at day 0, 2, and 5 after exposure to T‐cell/CD3xHER2 bsAbs mixture. The white dotted circle indicates tumoroid area. Green = BT474 WT, Lifeact‐EGFP‐transduced; blue = BT474 HER2 KO, Hoechst33342‐labeled; yellow = T‐cells, CellTracker Deep Red‐labeled; red = dead cells, PI stained. Scale bar = 100 µm. (C) Absolute T‐cell number recruited to the tumoroids on day 2 after exposure to T‐cell/CD3xHER2 bsAb mixture as shown in (B). The graph represents three independent experiments, each performed with 1–2 co‐cultures per condition, with each co‐culture generated in an individual well. Mean ± SEM is shown. (D) Fold change in number of HER2^+^ and HER2^−^ cells in tumoroids at day 5 after exposure to T‐cell/CD3xHER2 bsAb mixture calculated relative to day 0. Values below 1 indicate loss of cells. The graph represents three independent experiments, each performed with two co‐cultures per condition, with each co‐culture generated in an individual well. Mean ± SEM is shown. Two‐way ANOVA followed by Dunnett's multiple comparisons test was performed. *p < 0.05; ***p < 0.001.

Established HER2^+^, HER2^−^, and mosaic tumoroids were exposed to a mixture of T‐cells and CD3xHER2 bsAbs (Figure 2A), and T‐cell recruitment and tumoroid killing were monitored. At day 2, the number of T‐cells recruited to the tumoroids decreased as the proportion of HER2^+^ cells in the tumoroids decreased, corroborating that T‐cell recruitment required bsAb binding to the TAA (Figure 2B,C). No significant tumor killing was detected on day 2 in HER2^+^, HER2^−^, or mosaic tumoroids. On day 5, HER2^+^ cells were almost completely eliminated in all tumoroids. Effective bystander killing of HER2^−^ cells was observed in tumoroids that initially contained at least 50% HER2^+^ cells at the start of the experiment. A further reduction in the proportion of HER2^+^ cells prevented complete tumor destruction through bystander killing in mosaic tumoroids (Figure 2B,D). Although the number of T‐cells recruited to mosaic tumoroids containing 50% HER2^−^ cells dropped by ∼half as compared with 100% HER2^+^ tumoroids, tumoroid killing was near complete in both cases (Figure 2B,D). As we have shown previously, T‐cells in the absence of bsAbs did not accumulate in‐ or cause cell death in TAA‐positive or TAA‐negative tumoroids, and bsAbs alone had no effect [28] (Figure S3).

This data indicated that bystander killing in mosaic tumors depends on the proportion of TAA‐positive cells, with complete T‐cell mediated tumor destruction in the context of CD3xHER2 bsAbs in tumors containing up to 50% HER2^−^ cells.

### 2.3 Determining the Reach of Bystander Killing That Is Compatible with Destruction of TAA^−^ Tumor Islands in a Mosaic Tumor

**FIGURE 3:**
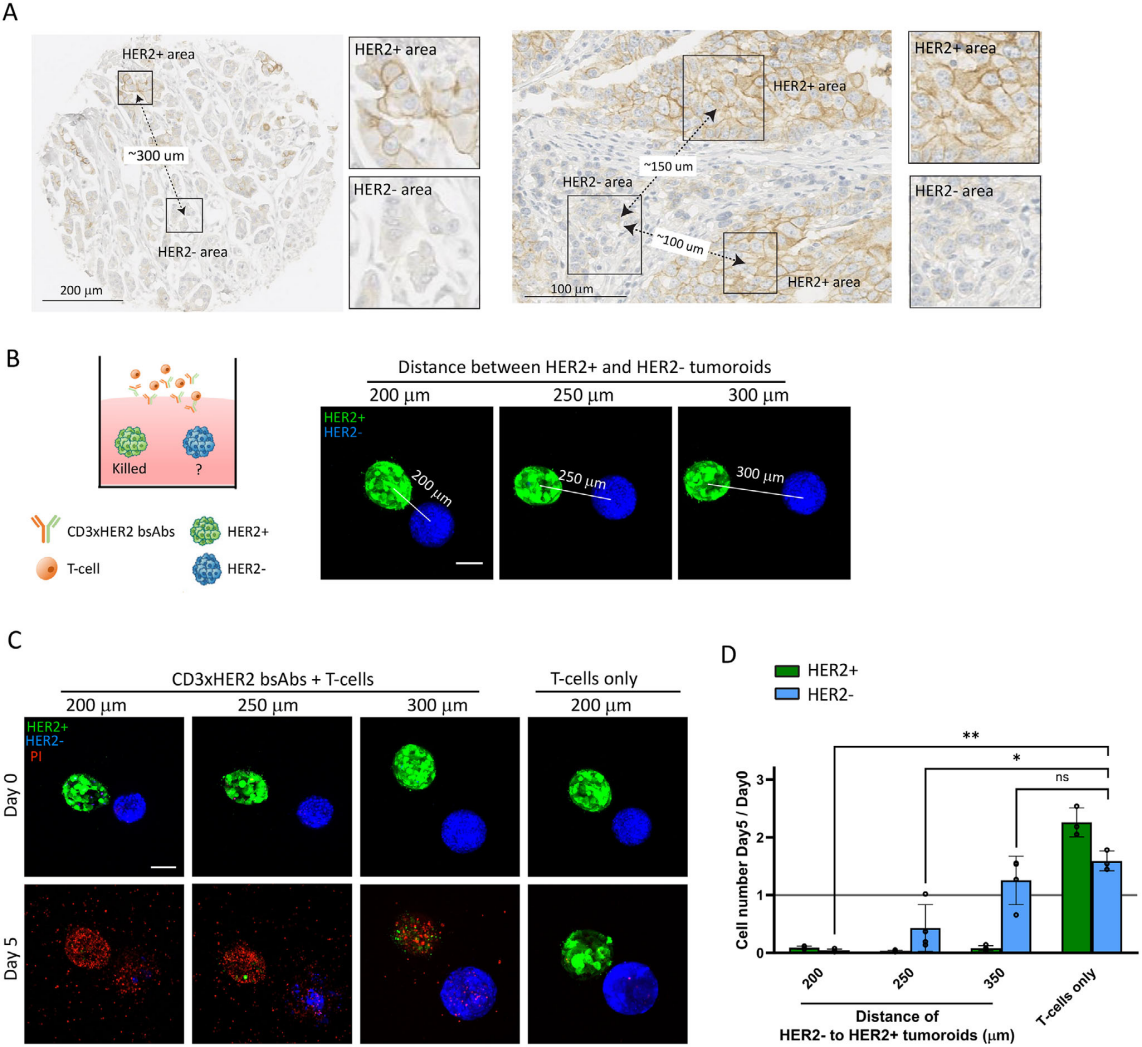
Determining the reach of bystander killing from HER2^+^ to HER2^−^ islands. (A) Representative images of HER2 IHC illustrating spatial separation of HER2^+^ (brown) and HER2^−^ islands in human breast cancer sections. Distances (center‐to‐center) between HER2^+^ and HER2^−^ islands (black squares) are indicated. Scale bar = 200 µm (left panel) or 100 µm (right panel) and smaller panels are zoomed in on boxed areas. (B) Left, a cartoon showing a schematic representation of the experimental setup to investigate the reach of bystander killing; right, representative 3D confocal images (maximum projections) showing co‐printed HER2^+^ (green) and HER2^−^ (blue) tumoroids spaced at distances between 200 µm and 300 µm (center‐to‐center) in collagen matrices. (C) 3D confocal imaging (maximum projections) of HER2^+^ and HER2^−^ tumoroids printed at the indicated distances before (day 0) and 5 days after exposure to T‐cell/CD3xHER2 bsAbs mixture compared with exposure to T‐cells alone. Green = BT474 WT, Lifeact‐EGFP‐transduced; blue = BT474 HER2 KO, Hoechst33342‐labeled; red = dead cells, PI stained. Scale bar = 100 µm. (D) Fold change in HER2^+^ and HER2^−^ cell numbers at day 5 after exposure to T‐cell/CD3xHER2 bsAb mixture calculated relative to day 0. Values below 1 indicate loss of cells. The graph represents three (T cells only) or four (T‐cell/CD3xHER2 bsAb mixture) independent experiments, each performed with one co‐culture. Mean ± SEM is shown. Two‐way ANOVA followed by Dunnett's multiple comparisons test was performed. ns = non‐significant; *p < 0.05; **p < 0.01.

HER2 intratumor heterogeneity may also present as spatially separated HER2^+^ and HER2^−^ islands within the tumor tissue as we observed in breast cancer patient tumors (Figure 3A). We adapted the model to mimic this situation (Figure 3B, cartoon). HER2^+^ and HER2^−^ tumoroids with an initial diameter of ∼150 µm were printed at distances varying between 200 and 300 µm, which reflected the sizes of and distances between islands observed in patient tumors (Figure 3A,B). The tumoroids were then exposed to a mixture of T‐cells and CD3xHER2 bsAbs for 5 days. Unexposed control tumoroids grew to a diameter of ∼200 µm. As anticipated, HER2^+^ tumoroids were eliminated by the treatment (Figure 3C,D). The efficacy of bystander killing of a HER2^−^ tumoroid in the neighborhood declined with increasing distance from HER2^+^ tumoroids. Complete bystander killing occurred at 200 µm, bystander killing was slightly reduced at 250 µm, and no significant bystander killing was observed at a 300 µm distance (Figure 3C,D). As a control, no killing of HER2^+^ and HER2^−^ tumoroids was observed in the absence of bsAbs.

This data indicated that the reach of bystander killing in the context of CD3xHER2 bsAbs is ∼250 µm for islands with a diameter of 150–200 µm. This setting represents a distribution observed in patient tumors, but reach will be influenced by in vivo tissue complexity.

### 2.4 Bystander Killing of TAA^−^ Tumors Is Not Associated with T‐cell Accumulation and Is Driven by Prior T‐cell Activation in TAA^+^ Neighboring Tumors

**FIGURE 4:**
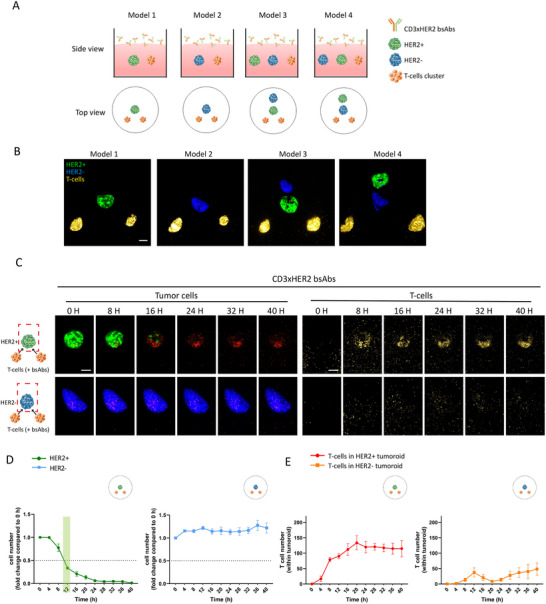
Experimental design to investigate spatial and temporal aspects of bystander killing. (A) Schematic representation of the experimental design (model 1–4) to investigate spatial and temporal aspects of bystander killing. (B) 3D confocal imaging (maximum projections) showing HER2^+^ tumoroids (green), HER2^−^ tumoroids (blue), and T‐cell clusters (yellow) co‐printed at specific positions in collagen matrices in the different models as indicated in (A). (C) 3D confocal imaging (maximum projections) showing tumor killing (left) and T‐cell recruitment (right) upon exposure to CD3xHER2 bsAbs for Model 1 and 2 followed over a time course of 40 h (note the T‐cell clusters are below the imaged area). Green = BT474 WT, Lifeact‐EGFP‐transduced; blue = BT474 HER2 KO, Hoechst33342‐labeled; yellow = T‐cells, CellTracker Deep Red‐labeled; red = dead cells, PI stained. Scale bar = 100 µm. (D) Quantification of T‐cell mediated tumor killing upon exposure to CD3xHER2 bsAbs for Models 1 and 2 followed over 40 h. Graphics show fold change in HER2^+^ (left) and HER2^−^ cell numbers (right) relative to 0 h. The light green bar indicates a 50% reduction in HER2^+^ cell number. The graph represents two independent experiments, each performed with 1–2 co‐cultures, with each co‐culture generated in an individual well. Mean ± SEM is shown. (E) Quantification of T‐cell recruitment upon exposure to CD3xHER2 bsAbs for Models 1 and 2 followed over 40 h. Graphs show absolute T‐cell numbers within HER2^+^ (left) and HER2^−^ tumoroids (right). The graph represents two independent experiments, each performed with 1–2 co‐cultures, with each co‐culture generated in an individual well. Mean ± SEM is shown.

To study spatial and temporal aspects of bystander killing, HER2^+^ and HER2^−^ tumoroids were printed adjacent to T‐cell clusters in various patterns, exposed to CD3xHER2 bsAbs, and monitored by confocal microscopy over a period of up to 40 h at 4 h time intervals (Figure 4A,B). In model 1 where only HER2^+^ tumoroids were generated, a 50% loss of tumor cells was observed at 12 h post‐bsAb addition, and 12 h later the tumoroid was eliminated (Figure 4C,D). T‐cell recruitment to the tumoroid occurred a few hours before tumor killing was observed, with T‐cell accumulation in the tumoroid starting 4–8 h post‐bsAb addition and reaching a maximum at 20 h (Figure 4C,E). By contrast, in model 2 where only HER2^−^ tumoroids were generated, no tumor killing or T‐cell accumulation was observed in the tumoroid area although some T‐cells were detected as they moved through that area (Figure 4C–E).

**FIGURE 5:**
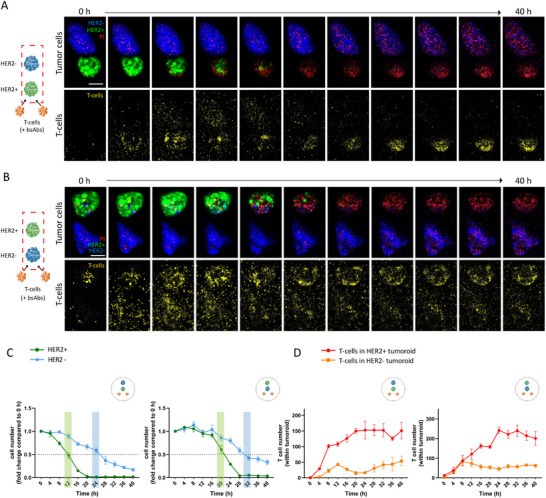
Spatiotemporal aspects of bystander killing after bsAb‐induced, T‐cell mediated antitumor cytotoxicity. (A, B) 3D confocal images (maximum projections) showing tumor killing (upper panel) and T‐cell recruitment (lower panel) upon exposure to CD3xHER2 bsAbs for Model 3 (A) and 4 (B) monitored over a time course of 40 h (note the T‐cell clusters are below the imaged area). Green = BT474 WT, Lifeact‐EGFP‐transduced; blue = BT474 HER2 KO, Hoechst33342‐labeled; yellow = T‐cells, CellTracker Deep Red‐labeled; red = dead cells, PI staining. Scale bar = 100 µm. (C) Quantification of T‐cell mediated tumor killing upon exposure to CD3xHER2 bsAbs for Model 3 (left) and 4 (right) followed over 40 h. Graphs show fold change in HER2^+^ and HER2^−^ cell numbers relative to 0 h. Light green bars indicate a 50% reduction in HER2^+^ cell number and light blue bars indicate a 50% reduction in HER2^−^ cell number. The graph represents two independent experiments, each performed with 1–2 (Model 3) or 2 (Model 4) co‐cultures, with each co‐culture generated in an individual well. Mean ± SEM is shown. (D) Quantification of T‐cell recruitment in HER2^+^ and HER2^−^ tumoroids upon exposure to CD3xHER2 bsAbs for Model 3 (left) and 4 (right) followed over 40 h. Graphs show absolute T‐cell numbers within HER2^+^ and HER2^−^ tumoroids. The graph represents two independent experiments, each performed with 1–2 (Model 3) or 2 (Model 4) co‐cultures, with each co‐culture generated in an individual well. Mean ± SEM is shown.

In model 3, HER2^+^ tumoroids were positioned at 200 µm from the T‐cell clusters, and HER2^−^ tumoroids were positioned at a further 200 µm distance. In this setup, killing of‐, and T‐cell recruitment to the HER2^+^ tumoroid showed identical kinetics as observed in model 1 (as expected based on the identical distance of the HER2^+^ tumoroid from the T‐cell clusters in these models) (Figure 5A,C,D). In the HER2^−^ tumoroid in model 3, 50% killing was reached after ∼24 h, lagging ∼12 h behind the HER2^+^ tumor without accumulation of T‐cells in the HER^−^ tumor (Figure 5A,C,D; Figure S4).

When the positions of HER2^+^ and HER2^−^ tumoroids were switched (model 4), a delay in the killing of the HER2^+^ tumoroids was observed. HER2^+^ tumoroid killing reached 50% at ∼20 h (as compared with 12 h in model 1) (Figure 5B,C). This was in line with a delay in T‐cell accumulation in the HER2^+^ tumoroid compared with model 3, and as expected based on the greater distance of the HER^+^ tumoroid from the T‐cell clusters (Figure 5B,D). In this setup, HER2^−^ tumoroid killing reached 50% at ∼32 h, approximately 12 h after HER2^+^ tumoroid killing. Again, no significant T‐cell accumulation occurred in the HER2^−^ tumor (Figure 5B–D; Figure S5).

These experiments further showed that bystander killing of HER^−^ tumor areas required prior bsAb‐mediated T‐cell activation in HER^+^ areas. Moreover, although the presence of some T‐cells in HER2^−^ tumoroids was not ruled out in all cases, the fact that no T‐cell accumulation was observed in TAA^−^ tumoroids indicated that bystander killing of TAA^−^ tumor areas does not involve direct local action of T‐cells.

### 2.5 Bystander Killing Involves Paracrine Signaling Through IFNγ and TNFα but Not T‐cell Interaction With TAA^−^ Tumor Areas

**FIGURE 6:**
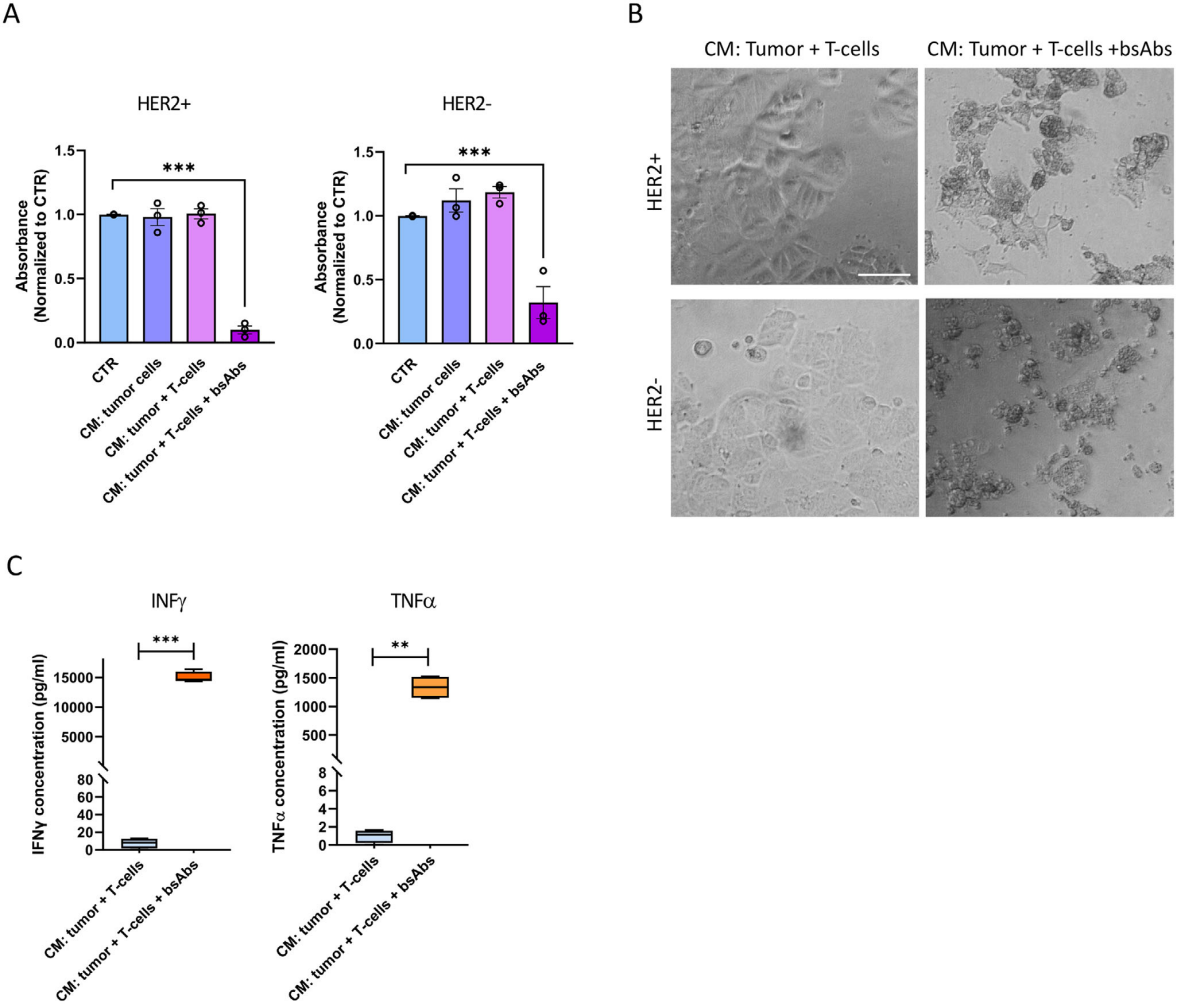
Soluble factors released during CD3xHER2 bsAb‐mediated T‐cell killing of HER2+ cells include cytokines and induce bystander killing. (A) Alamar Blue analysis of HER2^+^ (left) and HER2^−^ (right) cells 3 days after exposure to CM obtained from BT474 HER2^+^ cells in combination with T‐cells with or without CD3xHER2 bsAbs as indicated. Data are normalized to cell proliferation in a normal culture medium (CTR). The graph represents three independent experiments, each performed with 2D cultures in six wells of a 96‐well plate. Mean ± SEM is shown. Two‐way ANOVA followed by Dunnett's multiple comparisons test was performed. ***p < 0.001. (B) Transmission images showing HER2^+^ and HER2^−^ tumor cells exposed for 3 days to CM from the co‐culture of BT474 HER2^+^ tumor cells and T‐cells with or without CD3xHER2 bsAbs. Note cell rounding and debris in the presence of CM from co‐cultures with bsAbs indicating cytotoxicity. Scale bar = 50 µm. (C) ELISA analysis of IFNγ and TNFα levels in CM from 3‐day co‐cultures of BT474 HER2^+^ cells and T‐cells with or without CD3xHER2 bsAbs. Data from two T‐cell donors are combined, each performed in duplicate. An unpaired two‐tailed *t*‐test was performed. **p < 0.01; ***p < 0.001.

To further address whether bystander killing of HER2^−^ tumor areas occurred without the need for T‐cell contact at these sites, we collected conditioned medium (CM) from co‐cultures of HER2^+^ cells and T‐cells with or without CD3xHER2 bsAbs. Exposure to CM from co‐cultures with CD3xHER2 bsAbs effectively blocked proliferation and induced cell death visible as cell rounding and detachment, in both HER2^+^ and HER2^−^ cells (Figure 6A,B). No T‐cells were present in the CM and we verified that neither the bsAbs alone nor supernatant from cell lysates from HER2^+^ cells stressed by UV exposure, affected tumor cell viability (Figures S6, S7). This suggested that soluble factors produced during T‐cell activation were responsible for triggering bystander killing.

**FIGURE 7:**
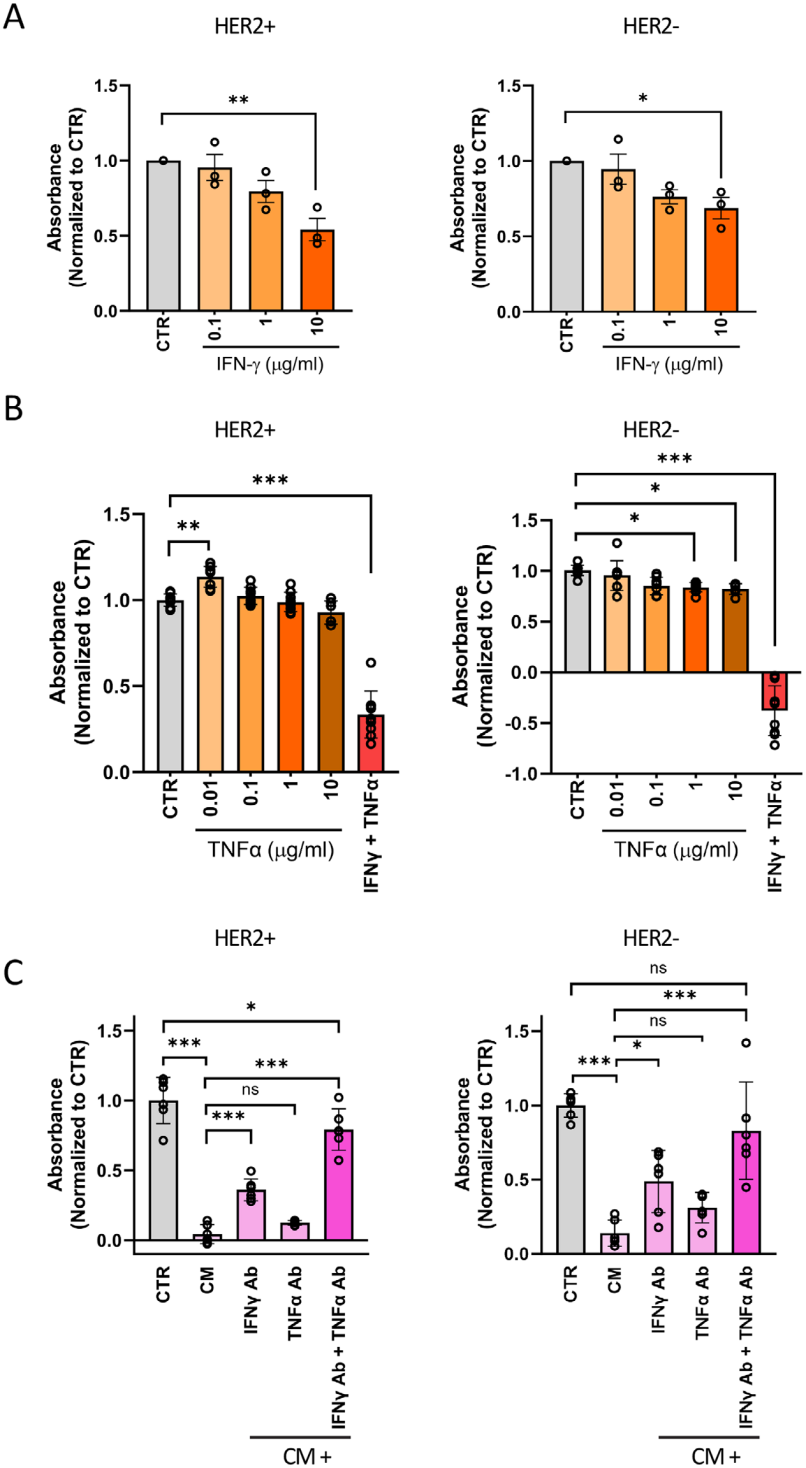
Bystander killing after bsAb‐induced, T‐cell‐mediated antitumor cytotoxicity involves paracrine signaling through IFNγ and TNFα. (A) Alamar Blue analysis of HER2^+^ and HER2^−^ cells 5 days after exposure to 0.1–10 µg/mL IFNγ. Data are normalized to cell proliferation without IFNγ treatment (CTR). The graph represents three independent experiments, each performed with 2D cultures in six wells of a 96‐well plate. Mean ± SEM is shown. Two‐way ANOVA followed by Dunnett's multiple comparisons test was performed. *p < 0.05; **p < 0.01. (B) Alamar Blue analysis of HER2^+^ and HER2^−^ cells 5 days after exposure to 0.01–10 µg/mL TNFα or a combination of 10 µg/mL IFNγ and 10 µg/mL TNFα. Data are normalized to cell proliferation without TNFα treatment (CTR). The graph represents three independent experiments, each performed with 2D cultures in three wells of a 96‐well plate. Mean ± SEM is shown. Two‐way ANOVA followed by Dunnett's multiple comparisons test was performed. *p < 0.05; **p < 0.01; ***p < 0.001. (C) Alamar Blue analysis of HER2^+^ and HER2^−^ cells 72 h after exposure to CM collected from the co‐culture of BT474 HER2^+^ tumor cells and T‐cells with CD3xHER2, with or without 10 µg/mL neutralizing Abs against IFNγ, TNFα, or a combination. Data are normalized to cell proliferation without treatment (CTR). Data with T‐cells from two donors are combined, each performed with 2D cultures in three wells of a 96‐well plate. Mean ± SEM is shown. Two‐way ANOVA followed by Dunnett's multiple comparisons test was performed. *p < 0.05; **p < 0.01; ***p < 0.001.

ELISA analysis revealed a robust and significant induction of IFNγ and TNFα in the CM from co‐cultures of HER2^+^ cells and T‐cells treated with CD3xHER2 bsAbs compared with the CM without bsAbs (Figure 6C). We next tested whether IFNγ and TNFα could mimic the cytotoxic activity of the CM from the co‐cultures. Exposure of HER2^+^ and HER2^−^ cells to IFNγ induced a concentration‐dependent increase in cytotoxicity, albeit less effective than the cytotoxicity caused by the CM (Figure 7A). Exposure to a concentration range of TNFα only slightly affected the growth of HER2^−^ cells, but the combination of IFNγ and TNFα was strongly cytotoxic (Figure 7B).

To address whether IFNγ and TNFα contributed to the cytotoxic effect of the CM, we used neutralizing antibodies to block these cytokines. The addition of an IFNγ neutralizing antibody partially blocked the cytotoxicity of the CM, while TNFα neutralization alone had no effect. However, the combination of IFNγ and TNFα neutralizing antibodies almost completely blocked cytotoxicity induced by the CM (Figure 7C).

**FIGURE 8:**
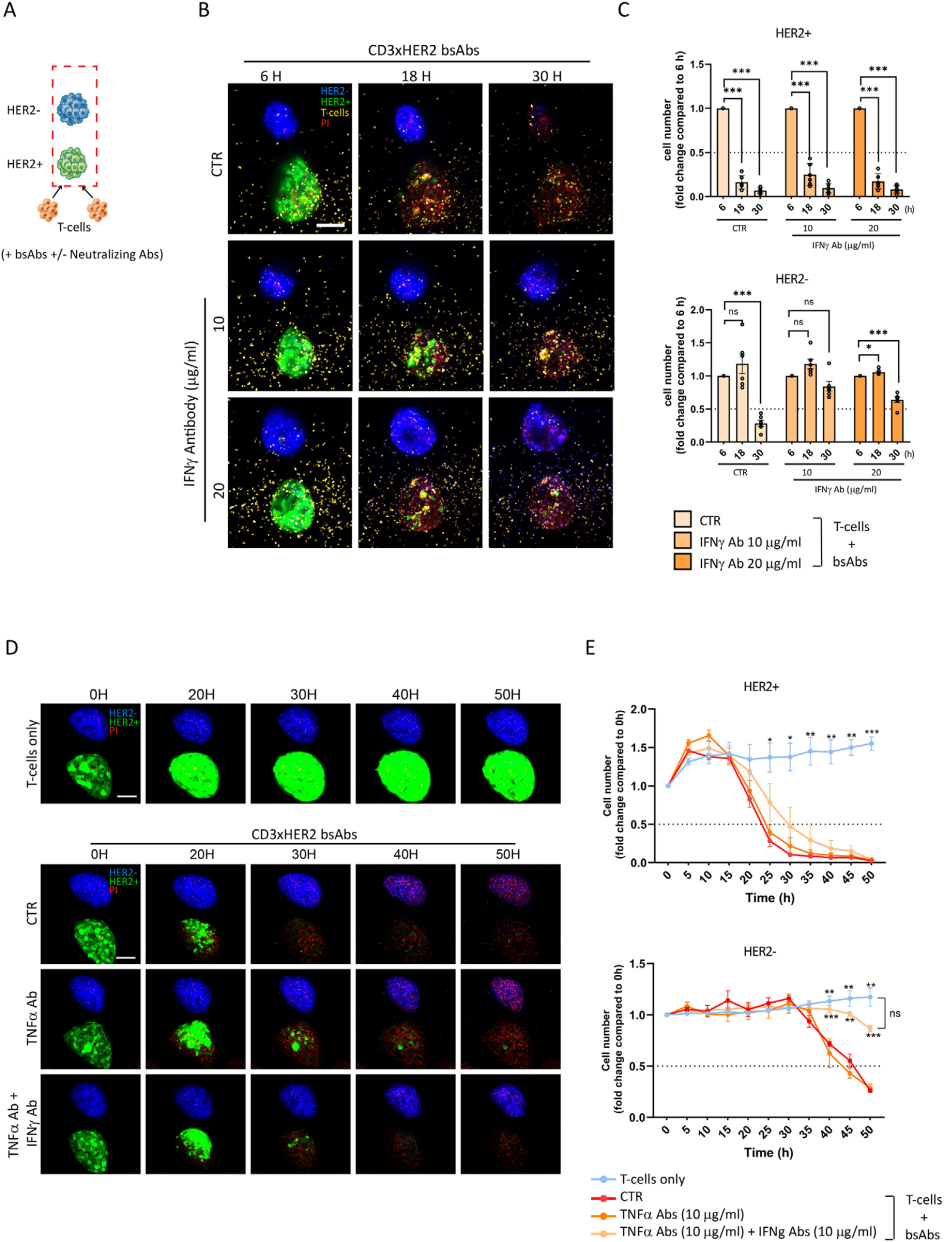
Neutralizing antibodies against IFNγ and TNFα block bystander killing of HER2‐ tumoroids after T‐cell‐mediated killing of HER2+ tumoroids via bsAbs. (A) Schematic representation of the experimental design based on Model 3 (see Figure 4A), with or without neutralizing antibodies. (B) Maximum projections of 3D confocal imaging showing HER2^+^ and HER2^−^ tumoroids co‐printed as described in (A) and treated with or without the indicated concentrations of an IFNγ neutralizing antibody. Images were taken 6, 18, and 30 h after exposure to CD3xHER2 bsAbs. Green = BT474 WT, Lifeact‐EGFP‐transduced; blue = BT474 HER2 KO, Hoechst33342‐labeled; yellow = T‐cells, CellTracker Deep Red‐labeled; red = dead cells, PI stained. Scale bar = 100 µm. (C) Quantification of T‐cell mediated tumor killing upon exposure to CD3xHER2 bsAbs in the presence or absence of IFNγ neutralizing antibody as shown in (B). Graphs show fold change in HER2^+^ and HER2^−^ cell numbers at the indicated time points relative to 6 h. The graph represents three independent experiments, each performed with 2 co‐cultures, with each co‐culture generated in an individual well. Mean ± SEM is shown. Two‐way ANOVA followed by Dunnett's multiple comparisons test was performed. ***p < 0.001. ns = non‐significant. (D) Maximum projections of 3D confocal imaging of HER2^+^ and HER2^−^ tumoroids co‐printed as described in (A) and treated with or without 10 µg/mL TNFα neutralizing antibody, or a combination of 10 µg/mL IFNγ and 10 µg/mL TNFα neutralizing antibodies. A co‐culture without bsAb treatment serves as a negative control. Images were taken over a 50 h time course (*note*: T‐cell signals were excluded from these images). Green = BT474 WT, Lifeact‐EGFP‐transduced; blue = BT474 HER2 KO, Hoechst33342‐labeled; red = dead cells, PI stained. Scale bar = 100 µm. (E) Quantification of T‐cell‐mediated tumor killing over time as shown in (D). Graphs show fold change in HER2^+^ and HER2^−^ cell numbers at the indicated time points relative to 0 h. The graph represents two independent experiments, each performed with 1–2 co‐cultures, with each co‐culture generated in an individual well. Mean ± SEM is shown. Two‐way ANOVA followed by Dunnett's multiple comparisons test was performed. *p < 0.05; **p < 0.01; ***p < 0.001 compared with CTR. ns = non‐significant.

Finally, we validated these findings in the 3D co‐culture setup (model 3) (Figure 8A). HER2^+^ tumoroids were eliminated by T‐cells in the presence of CD3xHER2 bsAbs and this was unaffected by neutralizing antibodies against IFNγ or TNFα (Figure 8B–E). By contrast, an IFNγ neutralizing antibody attenuated subsequent bystander killing of HER2^−^ tumoroids, with HER2^−^ tumoroids remaining alive up to at least 30 h, although the cell number slightly decreased (Figure 8B,C). As in earlier experiments, no T‐cell accumulation was observed in the HER2^−^ tumoroids (Figure 8B). A neutralizing TNFα antibody had no effect by itself. However, the combination of IFNγ and TNFα neutralizing antibodies completely blocked bystander killing of HER2^−^ tumoroids (without affecting the killing of HER2^+^ tumoroids), resulting in outcomes comparable to the T‐cell‐only control group for up to 50 h (Figure 8D,E).

These results showed that bystander killing triggered by initial CD3xHER2 bsAb‐mediated T‐cell cytotoxicity against HER2^+^ tumoroids occurs through paracrine signaling involving IFNγ and TNFα, and it does not require accumulation of T‐cells within HER2^−^ tumoroids.

## 3 Discussion

The presence of TAA^−^ tumor cells in solid tumors limits the efficacy of immunotherapies, allowing a subset of cancer cells to evade immune detection and cause tumor recurrence. However, the fact that bystander killing of TAA^−^ tumor cells has been reported in preclinical in vivo models for engineered TCR, CAR‐T, and bsAb anticancer immunotherapies, indicates that some TAA heterogeneity in patient tumors may be overcome [15, 16, 17, 18, 19, 20, 21, 22, 23]. Our work describes a quantitative assessment of the boundaries for bystander killing in the context of CD3xHER2 bsAbs using 3D ECM‐embedded tumoroids. We show that CD3xHER2 bsAb‐mediated interaction of T‐cells with a HER2^+^ tumor, triggers (1) bystander killing of up to 50% HER2^−^ cells in mixed areas and (2) IFNγ/TNFα‐mediated paracrine killing that reaches HER2^−^ tumor areas up to ∼250 µm distance in a 3D co‐culture setup. Importantly, we show that the bsAb‐mediated interaction of resting, nonactivated T‐cells with TAA^+^ cells in a tumor is sufficient to trigger such effective bystander activity.

Cytotoxic T‐cells eliminate target cells through the perforin‐granzyme B pathway and the Fas/FasL pathway [8, 9]. In addition, the activated T‐cells produce IFNγ, which diffuses and in mouse models has been shown to induce IFNγ receptor signaling in TAA^−^ bystander tumor cells in distant areas [13, 14]. Our finding that such paracrine effects of IFNγ can mediate bystander killing agrees with earlier studies demonstrating suppression of bystander killing by *IFNGR1* gene deletion or IFNγ neutralizing antibodies [23, 24]. The efficacy of this process in the context of resting T‐cells that are only activated by CD3xTAA bsAb‐mediated contact with 3D TAA^+^ tumor structures within an ECM‐containing TME has not been previously addressed.

A CRISPR screen has identified genes involved in IFNγ and TNFα signaling as critical mediators of CD8^+^ T cell‐mediated tumor cell killing [18]. In mouse models, both IFNγ and TNFα were found to be required for the killing of antigen‐loss variant tumor cells in established tumors [17]. Our experiments using neutralizing Abs and recombinant cytokines point to a more dominant role for IFNγ but show that both cytokines are indeed important for bystander killing in the context of bsAbs. The mechanism of action of IFNγ may involve direct IFNγ‐induced apoptosis of cancer cells [20] or stimulation of Fas expression on tumor cells [1]. In 2D co‐culture experiments, on‐target, as well as bystander killing mediated by CD3xCD19 or CD3xEGFR bsAbs was attenuated by genetic deletion or antibody blocking of Fas in the tumor cells [19, 22]. By contrast, in a similar experimental setup, bystander killing of 5T4 negative tumor cells mediated by CD3×5T4 bsAbs required IFNγ receptor but not Fas expression on the tumor cells [23]. Moreover, bystander killing of carcinoembryonic antigen (CEA)‐low colon cancer cells mediated by CD3xCEA bsAbs was suppressed by neutralizing antibodies targeting IFNγ but not those against FasL [24].

We do not rule out a role for Fas in CD3xHER2 bsAb induced bystander killing but the fact that we do not observe accumulation of T‐cells in TAA^−^ areas indicates that bystander killing in this case does not involve an interaction of FasL on T‐cells with Fas on the TAA^−^ tumor cells. Notably, others have reported for CD3xEGFR bsAbs that bystander killing does in fact rely on T‐cell interaction with TAA^−^ cells [22]. This may point to bsAb‐ or tumor cell type‐specific variations in the mechanisms of bystander killing. Our experiments show that T‐cells initially ignore HER2^−^ tumors and only after T‐cell activation in HER2^+^ tumors do we observe a subsequent killing of HER2^−^ tumors without T‐cell accumulation in those areas. This data, as well as our experiments using conditioned media from co‐cultures, indicate that soluble factors including IFNγ and TNFα that are secreted as T‐cells are activated in HER2^+^ areas, mediate bystander cytotoxicity of TAA^−^ tumor areas without direct T‐cell contact.

Using mixed co‐cultures of EGFR^−^ and EGFR^+^ tumor cells (not in ECM) with activated T‐cells, CD3xEGFR bsAbs have been shown to effectively trigger bystander killing when the proportion of EGFR^−^ tumor cells was up to 70% [22]. Our findings show that bystander killing triggered by resting T‐cells and CD3xHER2 bsAbs is fully effective for mixed tumors containing up to 50% HER2^−^ cells, and minimal bystander activity is observed in tumors containing 75% HER2^−^ cells. Although the CD3xEGFR and CD3xHER2 bsAbs may not be directly comparable, these similar outcomes suggest that (1) local activation of resting T‐cells by CD3xTAA bsAb‐mediated interaction with the positive tumor cells is sufficient to trigger maximal bystander killing, and (2) a tumor environment in which T‐cells have to migrate and cytokines have to diffuse through ECM and tumor tissue, does not impose a major limitation to the efficacy of this process.

In mouse models, bystander IFNγ signaling has been observed in TAA^−^ cells up to 800 µm away from the site of T‐cell mediated killing of a TAA^+^ tumor area [13]. This was achieved using engineered, activated T‐cells. In our setup using CD3xHER2 bsAbs, bystander killing of HER2^−^ tumors was observed up to 250 µm away from sites of T‐cell killing of HER2^+^ tumors. This smaller range of spreading may be due to the fact that we made use of resting, nonactivated T‐cells whose secretion of IFNγ and TNFα may be less prominent than that observed with engineered T‐cells. Alternatively, in vivo diffusion through tumor vasculature or the lifetime of cytokines may be more efficient than in the ECM scaffolds generated in our experimental setup. Notably, the limited reach of bystander killing in all these studies, suggests that bystander killing may increase efficacy of bsAbs, while toxicity due to bystander killing of nontumor cells will be restricted to the immediate vicinity of the tumor.

In conclusion, our findings indicate that bystander killing in the context of CD3xHER2 bsAbs involves initial bsAb‐mediated T‐cell cytotoxicity against HER2^+^ tumor areas and subsequent paracrine killing of HER2^−^ tumor areas through IFNγ and TNFα production by the activated T‐cells. Paracrine killing of HER2^−^ tumor areas does not appear to require local interaction of T‐cells with HER2^−^ cells, it can effectively eliminate up to 50% of TAA^−^ tumor cells in tumor areas where HER2^−^ and HER2^+^ cells are intermingled, and it can reach TAA^−^ tumor areas at significant distances in mosaic tumors.

## 4 Materials and Methods

### 4.1 Cell Culture and Reagents

Human breast cancer cell lines BT474 were obtained from the American Type Culture Collection (ATCC), authenticated using short tandem repeat (STR) profiling, and tested negative for mycoplasma prior to use. Cells were grown in RPMI 1640 (52400‐025, Gibco, Fisher Scientific, Landsmeer, The Netherlands) supplemented with 10% fetal bovine serum (FBS), 25 U/mL penicillin, and 25 µg/mL streptomycin in a humidified incubator with 5% CO_2_ at 37 °C. T‐cells were purified from Buffy coats of healthy donors (Sanquin, Amsterdam) by Ficoll Paque Plus (GE17‐1440‐02, Merck). CD3‐positive T‐cells were subsequently isolated using the Pan T‐cell Isolation Kit (130‐096‐535, Miltenyi Biotec) following the manufacturer's instructions as previously described [28]. T‐cells were collected and confirmed by CD3 flow cytometry before utilization. For 3D tumoroid—T‐cell co‐cultures, tumor cells were stained overnight at 37°C with 1 µg/mL Hoechst33342 (#610959; Thermo Fisher) and T‐cells were labeled with 1 µM CellTracker Deep Red (#C64565; Thermo Fisher). To label dead cells, 3D co‐culture media contained 0.4 µM propidium iodide (PI; P1304MP; Thermo Fisher). Cisplatin was obtained from the Pharmacy unit of the University Hospital, Leiden NL, and used as a stock concentration of 5 mg/mL to prepare a final concentration of 20 µg/mL in a culture medium. The CD3xHER2 bsAb used throughout this study at a final concentration of 1 µg/mL, was an Fc‐inert IgG1 CD3_wt_xHER2_169_ bsAb that was generated using controlled Fab‐arm exchange, termed DuoBody technology, which was described previously [28, 29, 30].

### 4.2 Lentiviral Transduction

Lentiviral particles were generated as previously described [31]. In short, Lenti‐X cells were transfected with lentiviral constructs and packaging vectors using polyethylenimine (408727; Sigma‐Aldrich). Target cells were transduced with viral supernatants 48 h later, using 8 µg/mL polybrene (H9268; Sigma‐Aldrich), and allowed to recover for 24 h posttransduction. BT474 cells were transduced with a pLenti‐Lifeact EGFP construct (#187686, Addgene) and sorted using a cell sorter (LE‐SH800SFP, SONY). To generate conditional HER2 knockout cells, BT474 cells were transduced with Lentiviral Edit‐R Cas9 plasmid (Dharmacon) and selected with 2 µg/mL blasticidin. Subsequently, inducible BT474‐Cas9 cells were transduced with a nontargeting sgRNA or either of two sgRNAs targeting the *ERBB2* gene (Sanger Arrayed Whole Genome Lentiviral CRISPR Library; KO1, *CCCCAGGGAGTATGTGAATGCCA*; KO2, *CAACTACCTTTCTACGGACGTGG*; Sigma‐Aldrich) and bulk selected with 4 µg/mL puromycin. Deletion of HER2 was induced by exposure of cells to doxycycline for 7 days followed by negative fluorescence‐activated cell sorting (FACS) sorting using a HER2 antibody.

### 4.3 Western Blotting

BT474 and BT474 HER2 knockout cells were lysed with RIPA buffer containing a 1% protease/phosphatase inhibitor cocktail (PIC; P8340, Sigma‐Aldrich). Samples were separated by SDS‐polyacrylamide (10%) gel electrophoresis and transferred to polyvinylidene difluoride (PVDF) membranes (Millipore) followed by blocking with 5% BSA in Tris‐buffered saline with 0.05% Tween‐20. Membranes were incubated with primary antibodies against HER2 (1:500; MA5‐14057, Thermo Scientific; RRID: AB_10977723) or tubulin (1:1000; T9026, Sigma‐Aldrich; RRID: AB_477593) overnight at 4°C followed by Horseradish peroxidase (HRP)‐conjugated secondary antibodies (Jackson Immunoresearch, anti‐rabbit 111‐035‐003; RRID: AB_2313567; anti‐mouse 115‐035‐003; RRID: AB_10015289) for 1 h at RT, and imaged with enhanced chemiluminescence substrate mixture (ECL plus, Amersham, GE Healthcare, Chicago IL, USA). Blots were imaged using an Amersham Imager (GE, Healthcare Life Science, Chicago, IL, USA).

### 4.4 Flow Cytometry

Cells were collected and washed twice with PBS supplemented with 2%FBS and 1 mM EDTA. For HER2, cells were incubated with HER2 antibody (1:200; 2165T, Cell Signaling; RRID: AB_10692490) for 1 h at 4°C, washed with FACS buffer (PBS supplemented with 0.5 % BSA and 2 mM EDTA) and incubated with AlexaFluor‐647 conjugated anti‐rabbit secondary antibody (1:500; 111‐605‐003, Jackson; RRID: AB_2338072) for 30 min at 4°C. For CD3, T‐cells were incubated with Brilliant Violet 421TM labeled anti‐human CD3 antibody (317343; BioLegend, San Diego CA, USA; RRID: AB_2565848) for 20 min at 4°C. Cell suspensions were washed twice with FACS buffer and analyzed by flow cytometry (CytoFLEX, Beckman Coulter). Unstained cells were used as a negative control.

### 4.5 Immunofluorescence

Cells were fixed and permeabilized by incubation with 1% formaldehyde and 0.1% Triton X100 in PBS for 15 min, followed by a blocking step with 0.5% w/v BSA (Sigma Aldrich) in PBS for 30 min. Then, cells were incubated with a HER2 primary antibody (1:200; 2165T, Cell Signaling; RRID: AB_10692490) in 0.5% w/v BSA in PBS overnight at 4°C. The cells were washed three times in PBS supplemented with 0.5% BSA (BSA‐PBS) and subsequently stained with AlexaFluor‐488 conjugated anti‐rabbit secondary antibody (1:1000, Mol Probes, A11008) and Hoechst 33258 (1:10,000, Sigma Aldrich, 861405) and Rhodamine Phalloidin (0.05 µM) for 1 h at room temperature, followed by three washing steps with 0.5% BSA‐PBS. Cell preparations were imaged using a Nikon ECLIPSE Ti2 confocal microscope.

### 4.6 Immunohistochemistry

Formalin‐fixed paraffin‐embedded tumors scoring positive or negative for HER2 were collected from tissue microarrays of breast cancer patients who entered the Erasmus University Medical Center (Rotterdam, the Netherlands) for local treatment of their primary disease between 1985 and 2005 [32]. This study was approved by the Erasmus MC medical ethics committee (MEC 02–953, approved 11th of April 2002). Tissue microarrays were processed for HER2 immunohistochemistry (IHC) using the HercepTest Kit according to the manufacturer's protocol (Dako Agilent, SK00121‐2). Slides were counterstained with hematoxylin and dehydrated through graded alcohol and xylene and cover slides were mounted with Pertex.

### 4.7 Culture Proliferation and Cytotoxicity Assays

Cell proliferation was analyzed using Alamar Blue, Sulforhodamine B (SRB), or IncuCyte (S3) assays. Cells were seeded at 5000 cells/well in 96‐well plates and processed for analysis at different time points. For Alamar Blue assays, 1X Alamar Blue (DAL1025, ThermoFisher) was added to each well and incubated for 4 h at 37°C. Fluorescence was measured using a FlexStation, with absorbance readings taken at 570 nm and 600 nm to assess cell viability. For SRB assays, cells were fixed by adding 30 µL of 50% TCA (Trichloroacetic acid) for 60 min at 4°C, then washed five times with deionized water, and allowed to dry. Next, the cells were stained with 60 µL of 0.4% SRB (S1402, Sigma) for 30 min at room temperature, washed four times with 1% acetic acid to remove unbound SRB, and air‐dried. The SRB‐bound protein was extracted by adding 150 µL of 10 mM Tris and measured at 540 nm using a plate reader (Tecan Infinite M1000). For IncuCyte assays, cells were placed in the IncuCyte and monitored using IncuCyte software (2020B).

For cytotoxicity, cells were seeded at 5000 cells/well in 96‐well plates overnight, followed by exposure to conditioned media (CM), recombinant cytokines, or cell lysates for 72 h. CM were collected 48 h after tumor‐T‐cell co‐culture with or without bsAbs and filtered to remove cells (431220, Corning). Cytokines were depleted from CM using neutralizing Abs against IFNγ (AF‐285‐NA, R&D systems, RRID: AB_354445) or TNFα (MAB225‐100, R&D systems, RRID: AB_2204150). Recombinant cytokines included IFNγ (AF‐300‐02, Gibco) and TNFα (210‐TA, R&D systems). In some experiments, cultures were exposed to cell lysates instead of CM. To prepare cell lysates, 1 million BT474 cells were seeded in a 24‐well plate and exposed to UV light for 15 min to induce cellular stress. The cells were then harvested and subjected to sonication to release cellular contents. The lysate was subsequently centrifuged to remove cellular debris.

### 4.8 ELISA Assay

CM from tumor‐T‐cell co‐cultures in the absence or presence of bsAbs was collected. IFNγ or TNFα in the CM was detected using ELISA kits (IFNγ, RAB0222, Sigma‐Aldrich; TNFα ELISA kit, 430207, Biolegend), according to manufacturer's procedures.

### 4.9 ECM Embedded Tumor T‐Cell Co‐culture Models

Collagen type I solution (Corning; 354249; high protein) was diluted to 1 mg/mL in DMEM containing 0.1 M HEPES (H0887, Sigma‐Aldrich) and 44 mM NaHCO_3_ (stock 440 mM; 71630, Fluka). 30 µL of collagen solution per well was loaded into a 384‐well imaging plate (781091, Greiner) and polymerized for 1 h at 37°C. Subconfluent monolayers of tumor cells were trypsinized, filtered (04‐0042‐2317, Sysmex), and re‐suspended in 100 µL PBS containing 2% polyvinylpyrrolidone (PVP; P5288, Sigma‐Aldrich). Droplets of the PVP/cell suspension containing ∼5000 cells were printed into the collagen matrix at defined x‐y‐z positions, creating cell clusters with a diameter of ∼150 µm that were positioned ∼150 µm above the bottom of the wells using an image‐guided micro‐injection robot (Life Science Methods, Leiden, NL) as previously described [29, 33, 34]. To create mosaic tumoroids, pLenti‐Lifeact EGFP‐transduced BT474 cells, and Hoechst‐stained BT474 HER2 knockout cells were mixed at different ratios before being printed into the collagen matrix. For tumoroid, T‐cell co‐cultures, approximately 50,000 CellTracker Deep Red‐labeled T cells, were added on top of the collagen matrix in the presence or absence of CD3xHER2 bsAbs as described previously [28]. Alternatively, T‐cells were suspended in a medium containing 2% PVP and printed into the collagen matrix using the same procedure as for the tumor cells.

### 4.10 Image Acquisition and Image Analysis

Tumoroids were imaged using a Nikon TE2000 confocal microscope equipped with a Prior automated stage controlled by NIS Element Software at 20× objective long‐distance objective in a temperature and CO_2_‐controlled incubator. Confocal Z stacks were generated through the entire z‐axis of the tumoroid taking an image every 10 µm. Images were captured 2 h after tumoroids were printed (day 0), and 2 and 5 days after the addition of bsAbs and T‐cells, or at 6, 18, and 30 h after the addition of bsAbs and neutralizing antibodies. For time‐lapse imaging, tumoroids were monitored for 40 h with a 4 h time interval or for 50 h with a 5 h time interval taking confocal images across the entire tumoroid z‐axis.

Automated image analysis was performed using ImageJ 1.53c and CellProfiler version 2.2.0. Images from the EGFP and Hoechst channels were pre‐processed using ImageJ to create masked images and to identify the boundaries of BT474 pLenti‐Lifeact EGFP and BT474 HER2 knockout tumoroids using watershed masked clustering (WMC) segmentation. Subsequently, the masked images, along with the other channels were imported into CellProfiler for further processing of each individual z‐plane as follows: (1) Identify Primary Objects: Identify and assign objects from the green channel (EGFP; HER2^+^ cells), the blue channel (Hoechst; HER2^−^ cells), and the far‐red channel (CellTracker Deep Red; T‐cells). Signals below 8 pixels in the green channel and below 5 pixels in the blue channel were filtered out to exclude dead cells, debris, and background noise; (2) mask objects: mask T‐cell images and remove all T‐cells outside the tumoroid boundary; (3) export the data and combine the information in each z‐plane for each tumoroid to quantify the total number of tumor cells and T‐cells per tumoroid.

### 4.11 Statistical Analysis

Statistical analyses were performed in GraphPad Prism 8 using one‐way analysis of variance (ANOVA) or two‐way ANOVA with Dunnett's post hoc test unless otherwise specified. Data were presented as the mean and standard error of the mean (SEM). Statistical significance was considered when *p* < 0.05. Experiments were conducted using at least two biological replicates, each performed in triplicate unless otherwise specified. Details are provided in the figure legends.

## Author Contributions

Erik H.J. Danen conceived and supervised the project. Chen‐Yi Liao, Elsa Neubert, and Erik H.J. Danen conceptualized and designed experiments. Chen‐Yi Liao and Michiel van Dijk performed experiments. Chen‐Yi Liao analyzed the data. Patrick Engelberts provided bsAbs, advice, and support. Annemieke Timmermans and John W. M. Martens generated IHC samples. Chen‐Yi Liao, Elsa Neubert, and Erik H.J. Danen wrote the manuscript. All authors read and reviewed the manuscript.

## Conflicts of Interest

This research was partly funded by Genmab B.V. Patrick Engelberts is an employee at Genmab BV and has ownership interests (including stocks, warrants, patents, etc.).

### Peer Review

The peer review history for this article is available at https://publons.com/publon/10.1002/eji.202451589

## Supporting information



Supplementary Materials

## Data Availability

All data generated or analyzed during this study are included in this published article and its supplementary information files.
