# Supplementary material for: CD3xHER2 bsAb‐Mediated Activation of Resting T‐cells at HER2 Positive Tumor Clusters Is Sufficient to Trigger Bystander Eradication of Distant HER2 Negative Clusters Through IFNγ and TNFα
Source: Eur J Immunol. 2025 Apr 3;55(4):e202451589. doi: 10.1002/eji.202451589 (PMC11967296; doi:10.1002/eji.202451589)
Supplement: Supplementary file 1 — Supplementary Materials [file EJI-55-e202451589-s001.pdf]

Supplementary Data

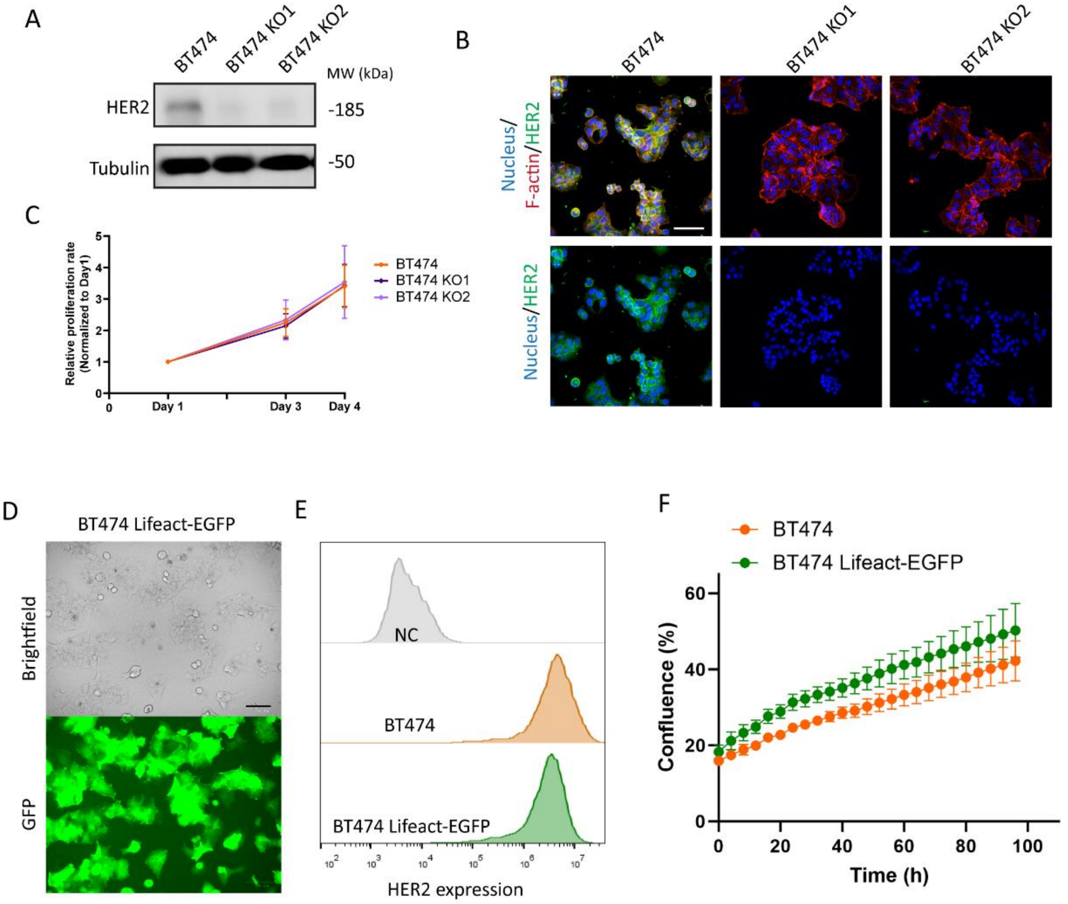

Supplementary Figure 1. Characterization of HER2 KO and Lifeact-EGFP expressing BT474 models. (A,B) Western blot (A) and immunofluorescence staining (B) showing loss of HER2 in two HER2 KO BT474 models. (A) Tubulin serves as loading control. (B) blue = Hoechst33342; red = Phalloidin; green = HER2 antibody. **(C)** SRB analysis of WT BT474 and two BT474 HER2 KO models. Data were normalized to day 1. Graph represents 3 independent experiments, each performed with 2D cultures in 6 wells of a 96-well plate. Mean ± SEM is shown. **(D)** Bright field and fluorescence images showing BT474 cells transduced with Lifeact-EGFP. Bar = 100 µm. **(E)** Flow cytometry showing HER2 expression in BT474 and BT474 Lifeact-EGFP transduced cell lines. NC, no primary antibody. **(F)** Quantification of proliferation of BT474 and BT474 Lifeact-EGFP transduced cell lines using IncuCyte. Graph represents 3 independent experiments, each performed with 2D cultures in 6 wells of a 96-well plate. Mean ± SEM is shown.

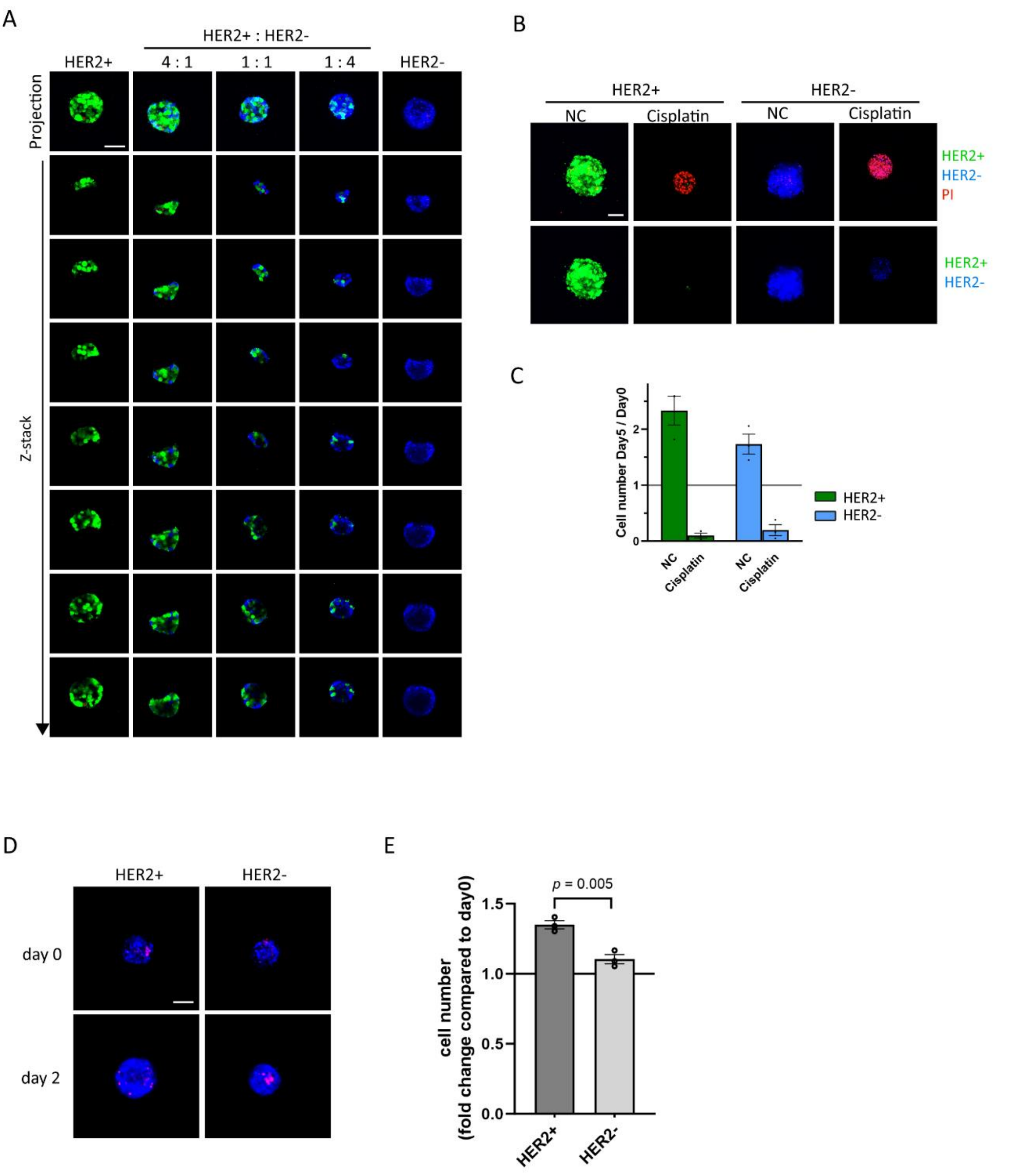

Supplementary Figure 2. Quantitative image analysis for viability of HER2^{+} and HER2^{-} cells in mixed tumoroids. (A) Maximum projection and z-stack images showing HER2^{+} (BT474 WT) and HER2^{-} (HER2 KO1) tumoroids, and mixed tumoroids containing the indicated ratios printed in collagen matrix. **(B)** Maximum projection images showing HER2^{+} and HER2^{-} tumoroids at day 5 with or without exposure to cisplatin during the last 24 hours. PI was added to the medium to label dead cells. Green = BT474 WT, Lifeact-EGFP-transduced; blue = BT474 HER2 KO, Hoechst33342-labeled; red = dead cells, PI staining. Bar = 100 µm. **(C)** Fold change in HER2^{+} and HER2^{-} cell numbers at day 5 relative to day 0 as shown in (B). Graph represents 3 independent experiments, each performed with one co-culture per condition. Mean ± SEM is shown. **(D)** Maximum projection images showing HER2^{+} and HER2^{-} tumoroids

both labelled with Hoechst33342 in 3D collagen matrix on day 0 and day 2. PI was added to label dead cells. Blue = Hoechst33342; red = PI. Bar = 100 µm. Note limited PI staining in all images under these CTR conditions. **(E)** Fold change in numbers of $HER2^{+}$ and $HER2^{-}$ cells in tumoroids at day 2 calculated relative to day 0 derived from image data shown in (D). Values above 1 indicate cell proliferation. Graph represents 3 independent experiments, each performed with one co-culture. Mean ± SEM is shown. An unpaired two-tailed t-test was performed. Note slight growth reduction for $HER2^{-}$ tumoroid also when both cell types were labeled with Hoechst33342.

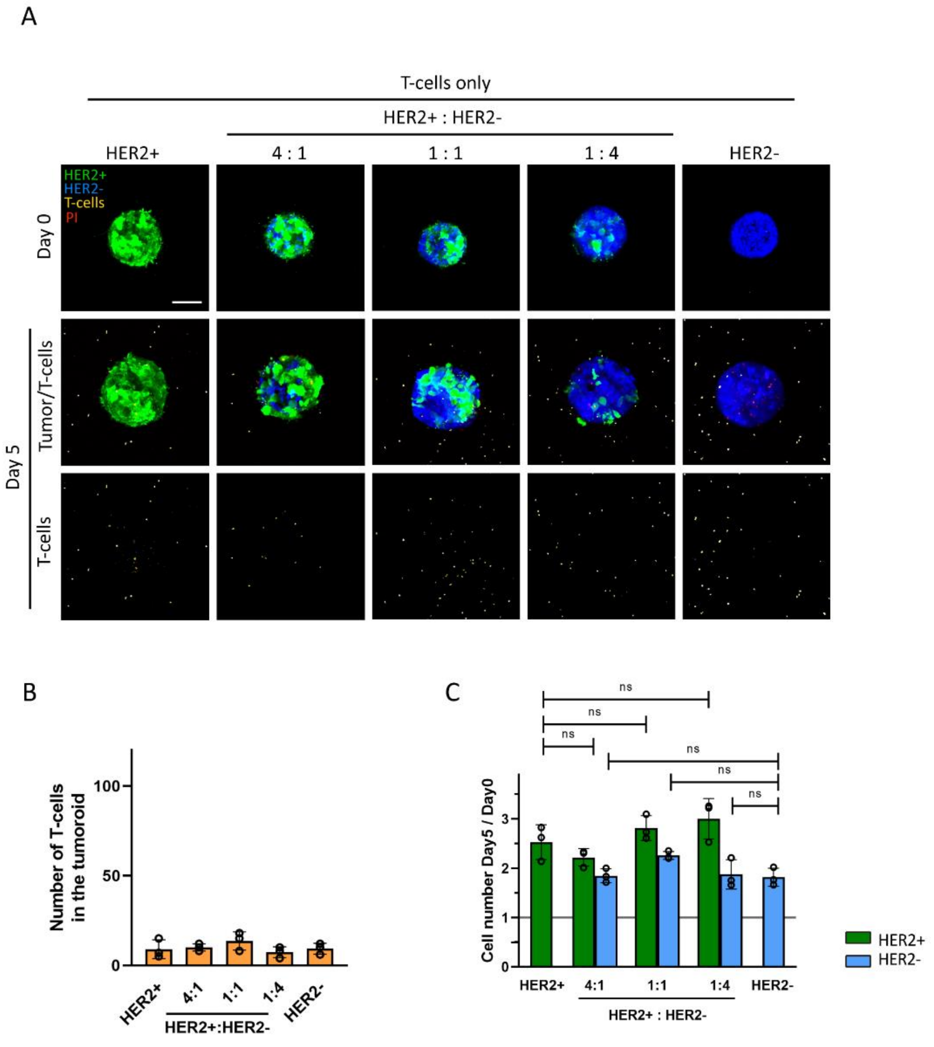

Supplementary Figure 3. No cytotoxic effect of T-cells in absence of bsAbs. (A) Maximum projection images showing HER2^{+} and HER2^{-} and mosaic tumoroids exposed to T-cells. Images taken on day 0 and day 5. Green = BT474 WT, Lifeact-EGFP-transduced; blue = BT474 HER2 KO, Hoechst33342-labeled; yellow = T-cells, CellTracker Deep Red-labeled; red = dead cells, PI staining. Bar = 100 µm. **(B)** Quantification of the number of T-cells recruited to the tumoroids on day 5. Graph represents 3 independent experiments, each performed with one co-culture per condition. Mean ± SEM is shown. **(C)** Fold change in HER2^{+} and HER2^{-} cell numbers at day 5 relative to day 0 as shown in (A). Graph represents 3 independent experiments, each performed with one co-culture per condition. Mean ± SEM is shown. Two-way ANOVA followed by Tukey's multiple comparisons test was performed. ns, non-significant.

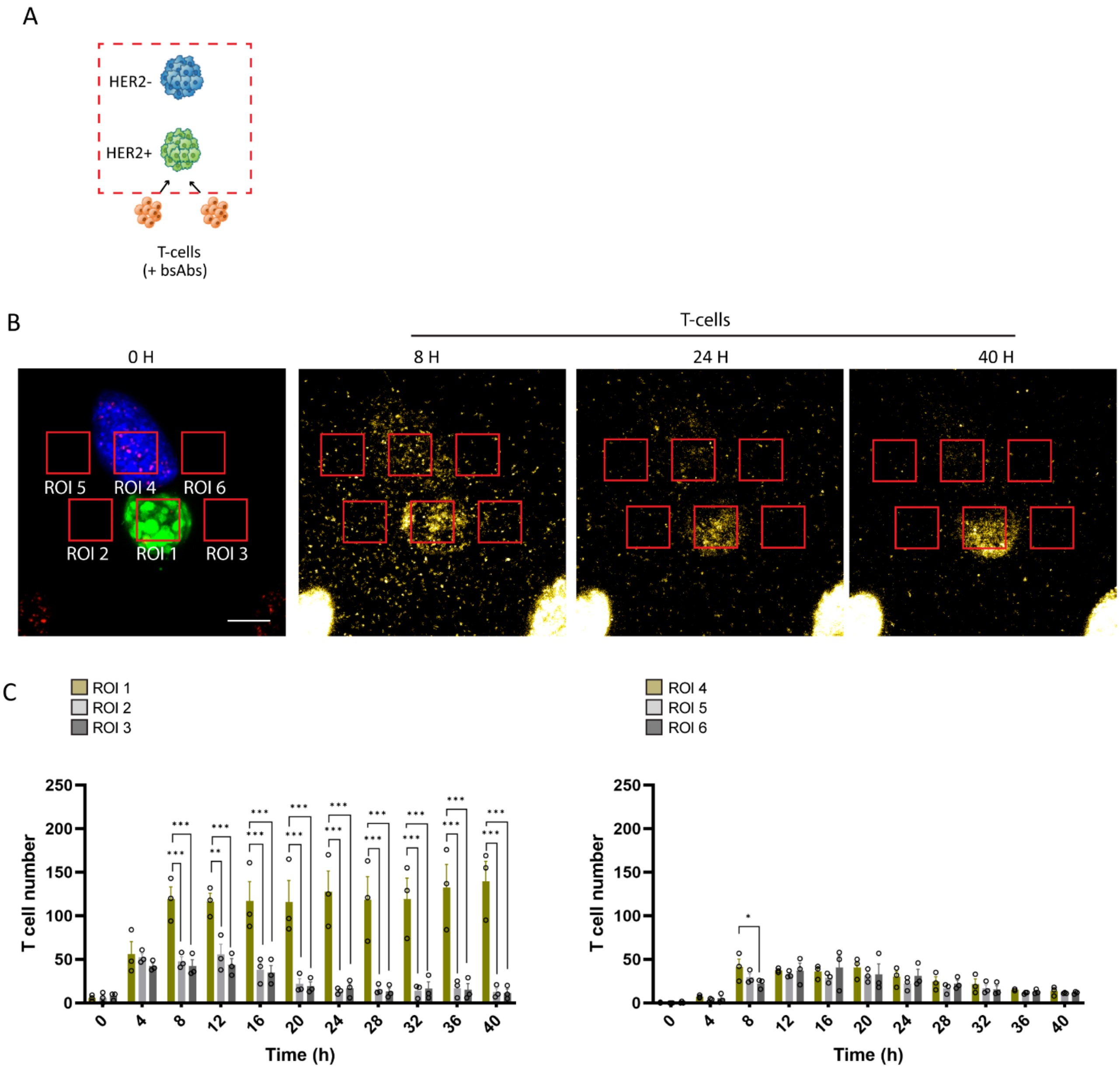

Supplementary Figure 4. Analysis of T-cell dynamics in Model 3. (A) Cartoon showing experimental model and area shown in (B). **(B)** 3D confocal imaging showing HER2^{+} (green) and HER2^{-} tumoroid (blue) positioned as shown in (A) and regions of interest (ROIs) analyzed for quantification of T-cell numbers over time. **(C)** Quantification of T-cell numbers in ROIs indicated in (B) over time. Graph represents data from 2 independent experiments, each performed in 1-2 replicates. Mean ± SEM is shown. Two-way ANOVA followed by Dunnett's multiple comparisons test was performed. *P < 0.05; ***P < 0.001.

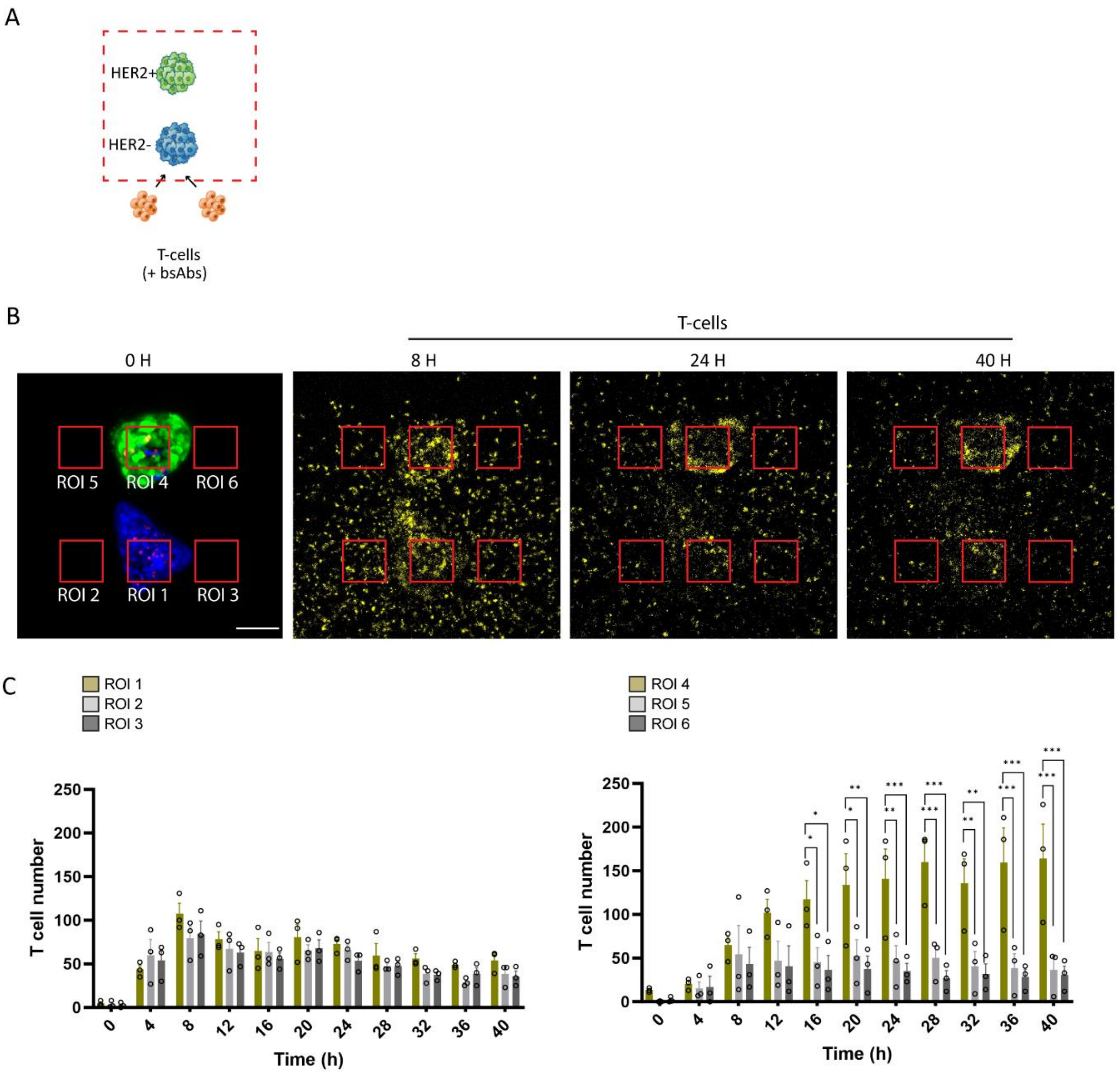

Supplementary Figure 5. Analysis of T-cell dynamics in Model 4. (A) Cartoon showing experimental model and area shown in (B). **(B)** 3D confocal imaging showing HER2⁻ tumoroid (blue) and HER2⁺ (green) positioned as shown in (A) and regions of interest (ROIs) analyzed for quantification of T-cell numbers over time. **(C)** Quantification of T-cell numbers in ROIs indicated in (B) over time. Graph represents data from 2 independent experiments, each performed in duplicate. Mean ± SEM is shown. Two-way ANOVA followed by Dunnett's multiple comparisons test was performed. *P < 0.05; **P < 0.01; ***P < 0.001.

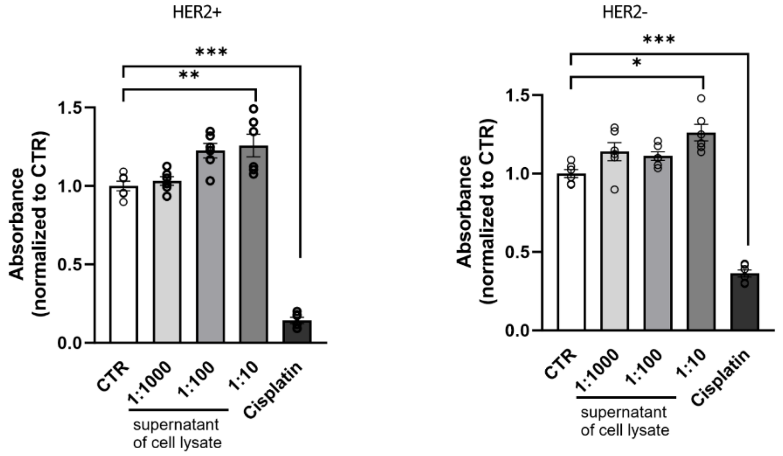

Supplementary Figure 6. Effect of soluble content released from stressed tumor cells on tumor cell viability. Alamar Blue analysis of $HER2^+$ and $HER2^-$ cell viability after 72 hours exposure to varying dilutions of lysates cleared from cell debris by centrifugation, derived from $HER2^+$ cells that were stressed by UV treatment. Untreated (CTR) and cisplatin (20 μg/ml) treated cells serve as controls.

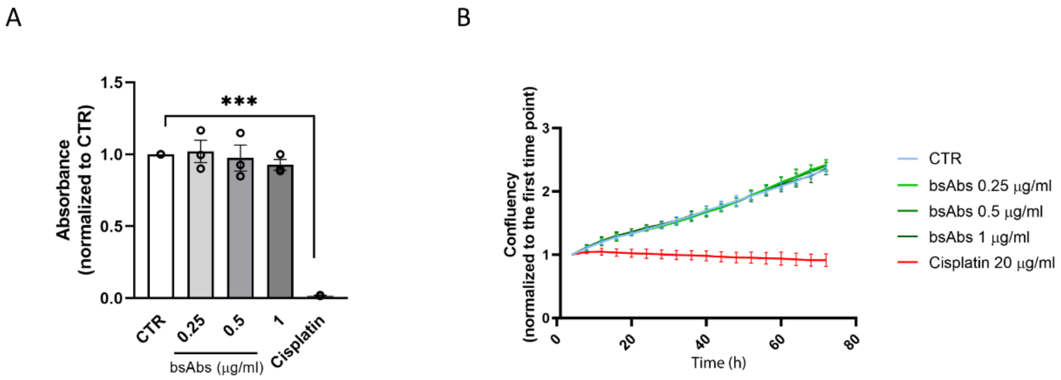

Supplementary Figure 7. BsAbs alone do not affect BT474 cell viability. (A) Alamar Blue analysis of BT474 cell viability 72 hours after treatment with varying concentrations of CD3xHER2 bsAbs. 20 µg/ml cisplatin serves as positive control cytotoxic agent. Data are normalized to cells grown in normal culture medium (CTR). Graph represents 3 independent experiments, each performed with 2D cultures in 6 wells of a 96-well plate. Mean ± SEM is shown. Two-way ANOVA followed by Dunnett's multiple comparisons test was performed. ****P* < 0.001. **(B)** Incucyte analysis showing expansion of BT474 cultures in absence or presence of different concentrations of CD3xHER2 bsAbs. 20 µg/ml cisplatin serves as positive control cytotoxic agent. Data are normalized to the first time point. Graph represents 3 independent experiments, each performed with 2D cultures in 6 wells of a 96-well plate. Mean ± SEM is shown.
